# Which is the optimal choice in lipid-lowering therapy for reducing major cardiovascular events? a network meta-analysis

**DOI:** 10.3389/fphar.2025.1626681

**Published:** 2025-09-03

**Authors:** Qiang Niu, Qilei Wang, Feng Chen, Bo Li

**Affiliations:** Department of Cardiology, Zibo Central Hospital, Zibo, China

**Keywords:** PCSK9 inhibitor, statin, MACE (major adverse cardiovascular events), LDL-C, inclisiran

## Abstract

**Background:**

Currently, there are various lipid-lowering therapies in clinical practice, and the emergence of PCSK9 inhibitors has undoubtedly added a valuable tool to lipid-lowering strategies. However, existing studies lack comparisons between different PCSK9 inhibitors and between the outcomes of using PCSK9 inhibitors alone versus in combination with statins. Therefore, this study aims to explore the efficacy and safety of different lipid-lowering regimens, aiming to determine the optimal lipid-lowering regimen.

**Methods:**

We searched PubMed/Medline and the Cochrane Library of Clinical Trials for related articles published up to October 2024. A network meta-analysis was applied including studies comparing PCSK9 inhibitors with statins or ezetimibe that have MACE outcomes. The efficacy endpoint was MACE, and the safety endpoints were new-onset diabetes and neurocognitive impairment.

**Results:**

We included a total of 29 studies, comprising 68,686 patients. Compared to oral lipid-lowering drugs, PCSK9 inhibitors combined with statins significantly reduced the incidence of MACE. While, PCSK9 inhibitors alone did not significantly reduce the risk of MACE.

**Conclusion:**

For patients requiring intensive lipid lowering, the combination of PCSK9 inhibitors with statins provides the greatest clinical benefit. While, for patients with moderate to low risk who need only single-drug therapy, statins are generally preferred over PCSK9 inhibitors considering various factors.

## Background

Atherosclerotic cardiovascular disease (ASCVD) remains a major threat to human life safety (Martin et al., 2024; Pirillo et al., 2023). Research on various risk factors of ASCVD remains a current hot topic. Blood lipid levels, particularly LDL-C, are widely recognized as independent risk factors closely associated with the occurrence and progression of atherosclerosis (Pirillo et al., 2023; Ference et al., 2024).

Currently, there are various lipid-lowering regimens, and statins have remained the cornerstone of lipid-lowering therapy due to robust evidence supporting their ability to reduce LDL-C and ASCVD risk (Collins et al., 2016). However, many patients, even when using the maximum tolerated dose of statins or adding other lipid-lowering drugs like ezetimibe, fail to achieve the ideal lipid levels recommended by guidelines (Serban et al., 2017).

While, the emergence of PCSK9 inhibitors may offer clinical promise to lipid-lowering therapy. By specifically binding to PCSK9, they prevent PCSK9 from binding to LDLR, inhibit PCSK9-mediated LDLR degradation, increase the number of LDLRs on the surface of hepatocytes, and thus significantly lower LDL-C levels and even further reduce the risk of major adverse cardiovascular events (MACE) (Shimada and Cannon, 2015). Numerous clinical trials have confirmed the potent LDL-C lowering effect of PCSK9 inhibitors (Sabatine et al., 2017; Schwartz et al., 2018; Raal et al., 2020). Consequently, more cardiovascular disease guidelines have recommended PCSK9 inhibitors; however, it is also noted that the recommended treatment regimens still involve the use of statins (Mach et al., 2020; Visseren et al., 2021; Grundy et al., 2019). That leads us to consider whether PCSK9 inhibitors can be used alone, without statins, to reduce the risk of MACE.

However, existing research lacks direct comparisons between different PCSK9 inhibitors and between monotherapy and combination therapy with statins. Therefore, this study is to explore the efficacy and safety of different lipid-lowering regimens in moderate to high-risk cardiovascular populations, including monotherapy with different PCSK9 inhibitors, combination therapy with different PCSK9 inhibitors and statins, and oral lipid-lowering drugs (statins or ezetimibe) alone, aiming to determine the optimal lipid-lowering regimen.

## Methods

### Guidance and protocol

The methodology for the network meta-analysis follows the PRISMA-NMA guidelines (Moher et al., 2009). The protocol of the present study was registered in Open Science Framework database (ID:CRD42024558622).

### Data sources and search strategy

We searched PubMed/Medline and the Cochrane Library of Clinical Trials for related articles published up to October 2024. The following keywords were used: PCSK9 inhibitor, alirocumab, evolocumab, inclisiran. Citations were screened at the title and abstract level and retrieved if considered relevant. Meanwhile, we checked the references of the retrieved studies for additional studies. The details of the search strategy conducted are presented in Table 1.

**TABLE 1:** Literature screening flow chart.

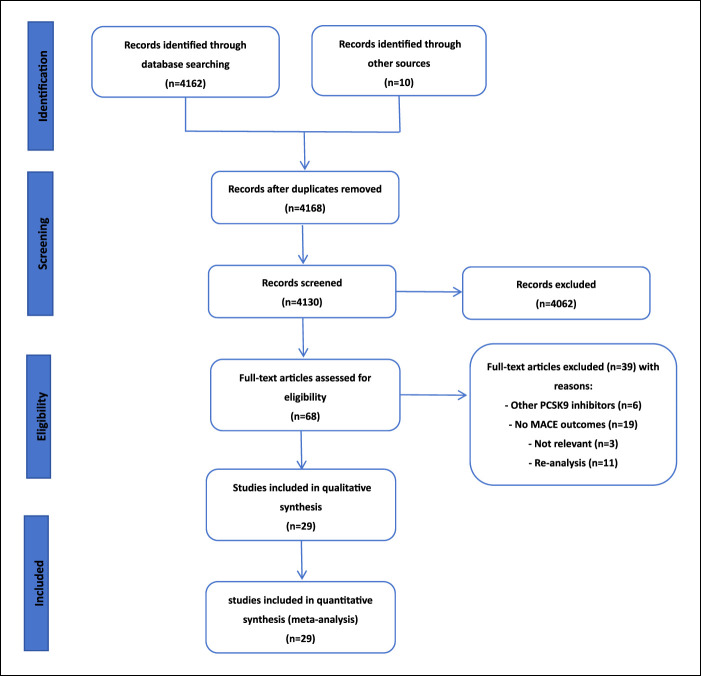

### Selection criteria

We only included randomized controlled trials (RCT) that met the following inclusion criteria: 1. The study population consisted of adult patients with dyslipidemia or diagnosed ASCVD; 2. The trial compared alirocumab, evolocumab, or inclisiran with control strategies (placebo and/or other lipid-lowering drugs); 3. The study must include at least one of the following outcomes: MACE (cardiovascular death, non-fatal myocardial infarction, stroke, re-intervention, rehospitalization), new-onset diabetes, or neurocognitive impairment. There were no restrictions on follow-up duration and study size. Observational studies (including single-arm trials), case reports, case series studies, meta-analyses, and studies with duplicate data were excluded from this analysis.

### Data extraction

Study selection was independently conducted by two authors (Niu Qiang and Wang Qilei). Based on titles and abstracts retrieved from electronic searches, irrelevant studies were eliminated. Subsequently, these two authors examined the full texts of the remaining studies and the full-text reports of all potentially relevant trials and assessed them independently for eligibility on the basis of the defined inclusion criteria. The corresponding author (Li Bo) was responsible for contacting to obtain missing information when assessing inclusion criteria or lacking critical data. Any discrepancies were resolved by discussion. The risk of bias in included studies was assessed according to Cochrane Collaboration guidelines.

### Quality assessment

We used the Cochrane risk of bias assessment tool (ROB 2) for assessing the risk of bias in eligible studies (Sterne et al., 2019). The grading of recommendations assessment, development, and evaluation (GRADE) were used to evaluate the certainty of evidence based on the risk of bias, inconsistency, indirectness, Imprecision, publication bias and incoherence (Puhan et al., 2014).

### Statistical analysis

Statistical analysis was performed using the gemtc package in R software (version 4.1). Bayesian network meta-analysis was conducted using a consistency model, compare the efficacy and safety of any two therapies to determine the optimal treatment plan. Dichotomous variables were represented as risk ratios (RRs) and 95% confidence intervals (CI) were estimated. These models were based on 30,000 iterations after an initial 10,000 iterations. We considered there were statistically significant when the 95% confidence intervals did not cross 1. The surface under the cumulative ranking curve (SUCRA) was used to estimate the probability of treatment drugs ranking for each outcome. Statistical heterogeneity was interpreted by the I2 statistic (value exceeding 50% was considered substantial) (Higgins and Thompson, 2002). Publication bias was assessed using funnel plots with Egger regression test if more than 10 trials were included (Egger et al., 1997).

## Results

### Search results and search results

**FIGURE 1:**
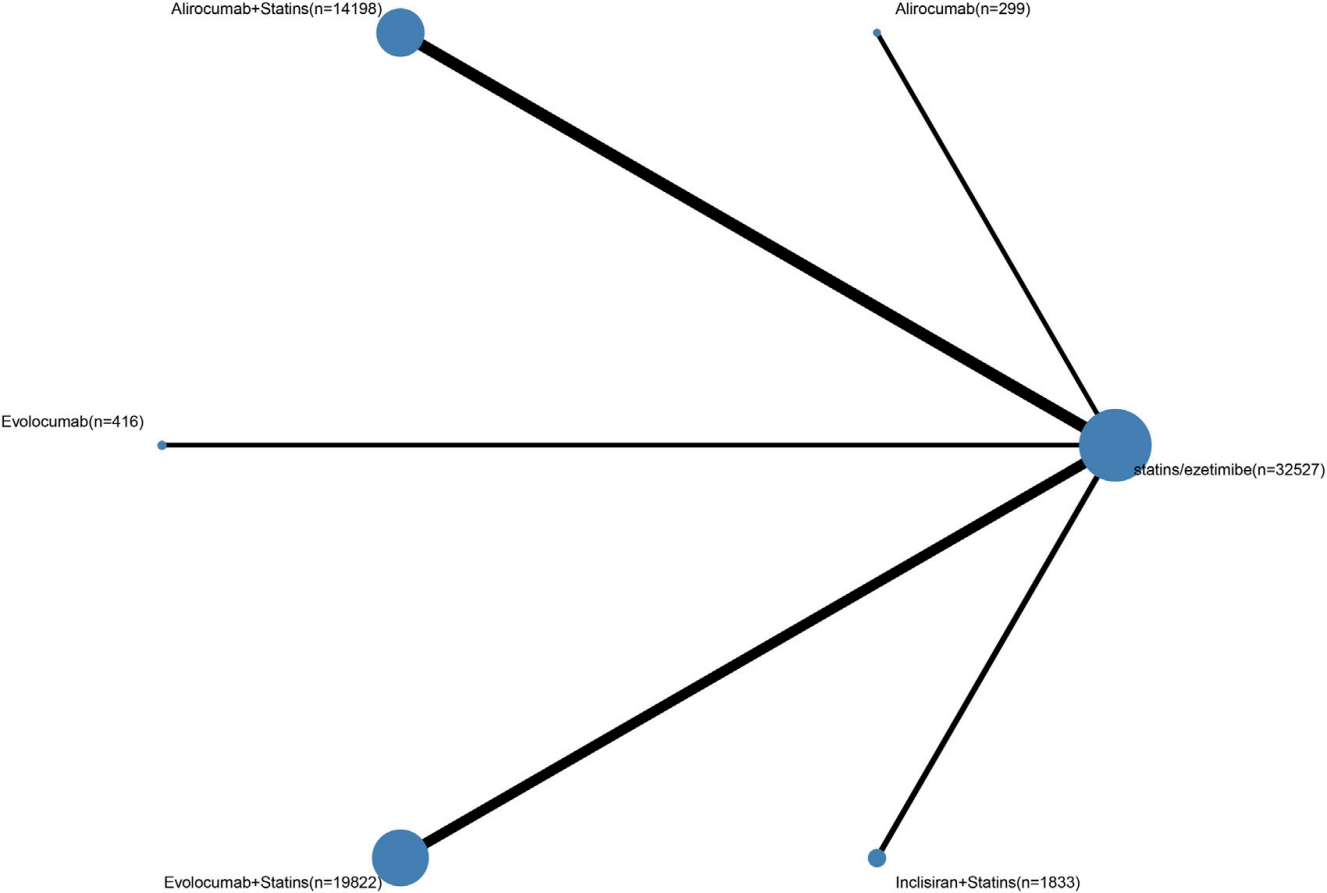
Network diagram of RCT.

We included a total of 29 studies involving 68,686 patients, comprising 14 studies on alirocumab, 12 studies on evolocumab, and 3 studies on inclisiran (Sabatine et al., 2017; Schwartz et al., 2018; Raal et al., 2020; Moriarty et al., 2015; Stroes et al., 2016; Kereiakes et al., 2015; Cannon et al., 2015; Ginsberg et al., 2016; Teramoto et al., 2016; Koh et al., 2018; Robinson et al., 2015; Bays et al., 2015; Farnier et al., 2016; Han et al., 2020; Räber et al., 2022; Janik et al., 2021; Nicholls et al., 2022; Hirayama et al., 2014; Blom et al., 2014; Nissen et al., 2016; Nicholls et al., 2016; Robinson et al., 2014; Raal et al., 2015; Kiyosue et al., 2016; Koskinas et al., 2019; Koren et al., 2012; Ray et al., 2020) (Figure 1). Among these studies, there were also 4 studies comparing monotherapy with PCSK9 inhibitors against control groups. Study sizes ranged from 107 to 27,564 participants, with average ages ranging from 50.6 to 66.1 years, and male proportions varying from 47.7% to 81.5%. All the 29 studies was judged to be high by using the ROB 2.0. The characteristics of the participants and trials are reported in Table 2.

**TABLE 2:** Baseline characteristics of included studies.

Trial	Register number	Sample size	Male (%)	Age, years	Diabetes (%)	Follow-up/Treatment duration	Investigational drug	Statin (%)
1.ODYSSEY ALTERNATIVE	NCT01709513	313	54.8	63.4	23.9	24W	Alirocumab	20.1
2.ODYSSEY CHOICE II	NCT02023879	231	56.3	63.8	16.5	24W	Alirocumab	0
3.ODYSSEY COMBO 1	NCT01644175	314	66.2	63	43.3	52W	Alirocumab	100
4.ODYSSEY COMBO 2	NCT01644188	720	73.6	61.6	31.0	52W	Alirocumab	99.9
5.ODYSSEY HIGH FH	NCT01617655	107	53.3	50.6	14	78W	Alirocumab	100
6. ODYSSEY JAPAN	NCT02017898	215	60.6	60.8	68.5	52W	Alirocumab	100
7.8. ODYSSEY KT	NCT02289963	199	70.1	60	41	24W	Alirocumab	100
9.ODYSSEY LONG TERM	NCT01507831	2,338	62.3	60.5	34.6	78W	Alirocumab	100
10.ODYSSEY OPTIONS I	NCT01730040	354	65.3	63.1	50	24W	Alirocumab	100
11ODYSSEY OPTIONS II	NCT01730053	305	61.3	60.9	41.3	24W	Alirocumab	100
12.ODYSSEY OUTCOMES	NCT01663402	18,924	74.8	58.6	28.8	208W	Alirocumab	100
13.ODYSSEY EAST	NCT02979015	612	75	58.6	27.5	24W	Alirocumab	100
14.PACMAN-AMI	NCT03067844	298	81	58.5	10.3	52W	Alirocumab	100
15.CANTAB	-	2,171	58.2	63	-	96W	Alirocumab	90
16.HUYGENS	NCT03570697	161	71.4	60.5	16.8	50W	Evolocumab	100
17.YUKAWA I	NCT01652703	307	62.9	61.5	38.1	12W	Evolocumab	100
18.DESCARTES	NCT01516879	901	47.7	56.2	11.5	52W	Evolocumab	87.7
19.FOURIER	NCT01764633	27,564	75.4	62.5	20	113W	Evolocumab	100
20.GAUSS III	NCT01984424	218	51.4	58.8	11.9	24W	Evolocumab	0
21.GLAGOV	NCT01813422	968	72.2	59.8	20.9	76W	Evolocumab	98.6
22.LAPLACE-2	NCT01763866	1896	54.2	59.8	15.5	78W	Evolocumab	100
23.OSLER 1 & 2	NCT01439880 NCT01854918	4,465	50.5	57.9	13.4	48W	Evolocumab	70.1
24.RUTHERFORD-2	NCT01763918	329	57.8	51.2	-	12W	Evolocumab	100
25.YUKAWA-2	NCT01953328	404	60.4	61.5	48.8	12W	Evolocumab	100
26. EVOPACS	NCT03287609	307	81.5	60.7	22.4	8W	Evolocumab	100
27.MENDEL	NCT01375777	406	34	50.8	0.2	12W	Evolocumab	0
28.ORION-9	NCT03397121	481	47.1	56	10	540D	Inclisiran	100
29.ORION-10	NCT03399370	1,561	69.4	66.1	45	540D	Inclisiran	90
30.ORION-11	NCT03400800	1,617	71.7	64.8	35.1	540D	Inclisiran	94

### Efficacy results

**FIGURE 2:**
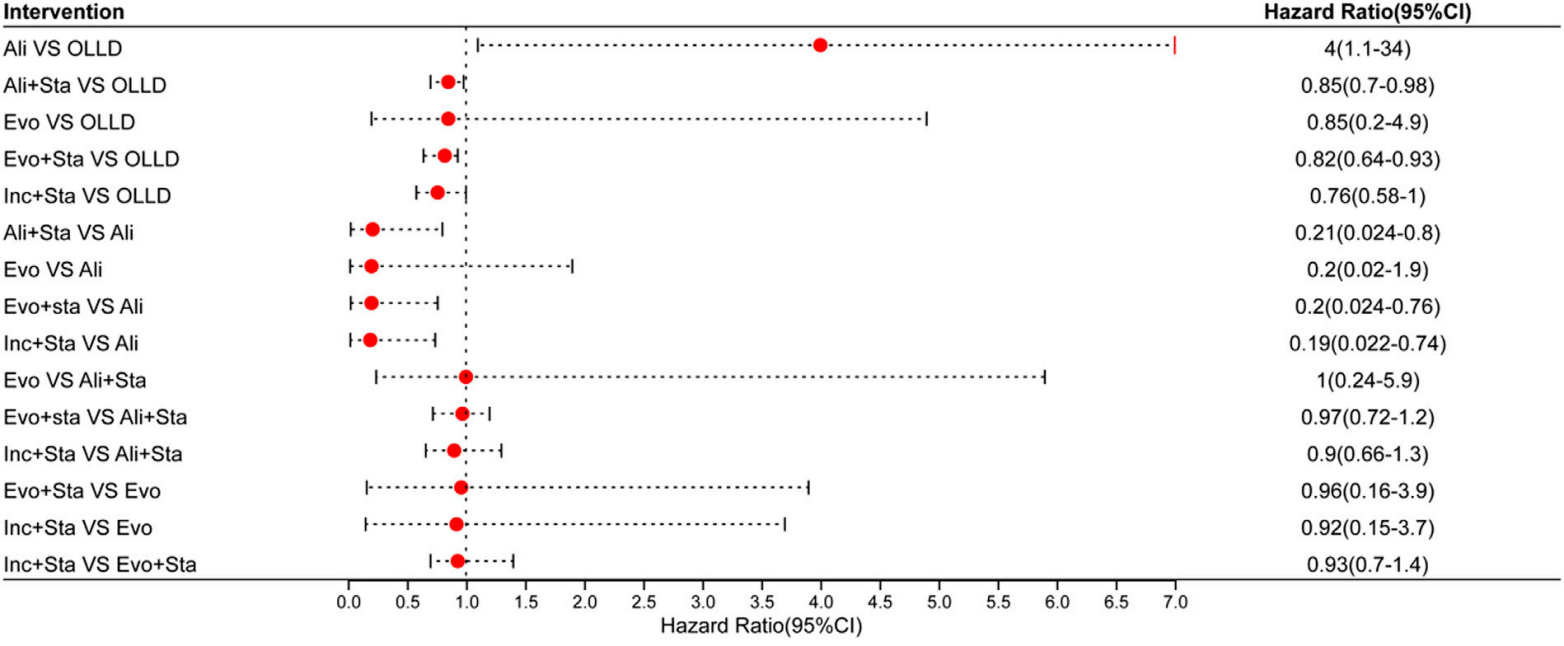
Ali: alirocumab; Evo: evolocumab; Inc: inclisiran; Sta:statins; OLLD: oral lipid-lowering drugs (statins, ezetimibe, or their combination).

**FIGURE 3:**
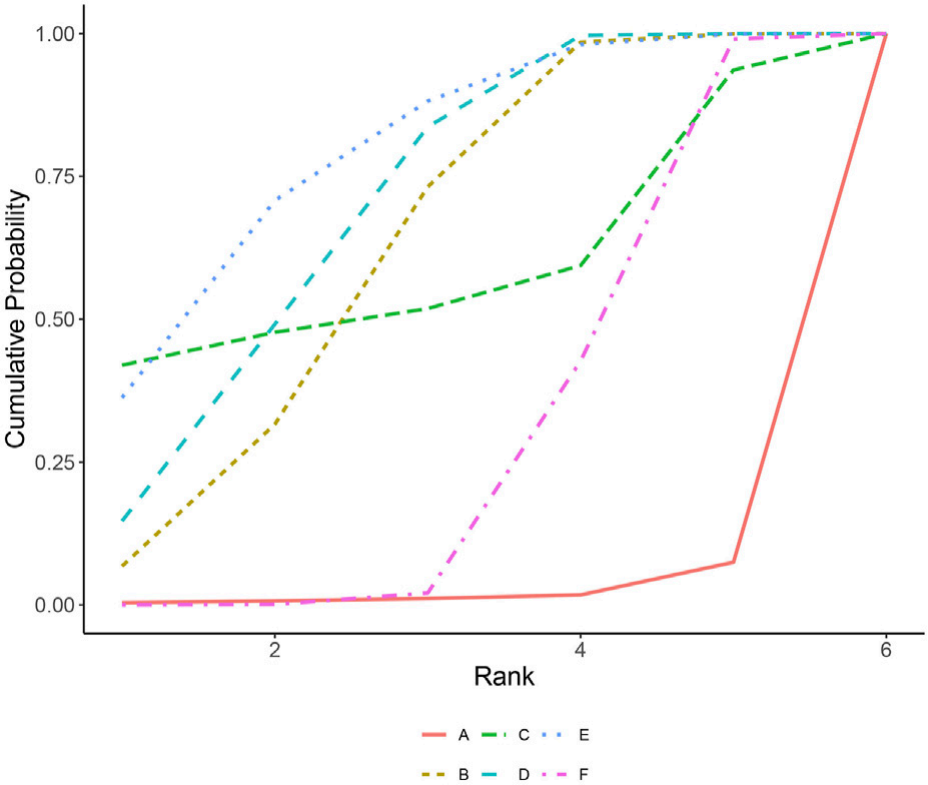
A: alirocumab; B: alirocumab combined with statins; C: evolocumab; D: evolocumab combined with statins; E: inclisiran combined with statins; F: oral lipid-lowering drugs.

All included studies reported occurrences of major adverse cardiovascular events (MACE). In terms of MACE incidence, PCSK9 inhibitors combined with statins showed significant reductions compared to oral lipid-lowering drugs (statins, ezetimibe, or their combination) (Figure 2), including alirocumab combined with statins (RR 0.85, 95% CI 0.7–0.98, high certainty); evolocumab combined with statins (RR 0.82, 95% CI 0.64–0.93, high certainty); and inclisiran combined with statins (RR 0.76, 95% CI 0.58–1, high certainty). Compared to the use of alirocumab alone, the combination of PCSK9 inhibitors with statins significantly reduces the incidence of MACE events (moderate to low certainty): alirocumab combined with statins (RR 0.21, 95% CI 0.024–0.8); evolocumab combined with statins (RR 0.2, 95% CI 0.024–0.76); inclisiran combined with statins (RR 0.19, 95% CI 0.022–0.74). However, monotherapy with PCSK9 inhibitors compared to oral lipid-lowering drugs not only didn’t show significant reductions but even increased the risk of MACE events (high certainty): alirocumab (RR 4, 95% CI 1.1–34, high certainty); evolocumab (RR 0.85, 95% CI 0.2–4.9, high certainty). The SUCRA values indicate the overall ranking probability of each treatment regimen for this outcome, with inclisiran combined with statins considered the best treatment regimen for reducing MACE risk, with an SUCRA value of 0.79 (Figure 3). The subsequent rankings were evolocumab combined with statins (0.69), alirocumab combined with statins (0.62), evolocumab monotherapy (0.59), oral lipid-lowering drugs (0.29), and alirocumab monotherapy (0.02).

**FIGURE 4:**
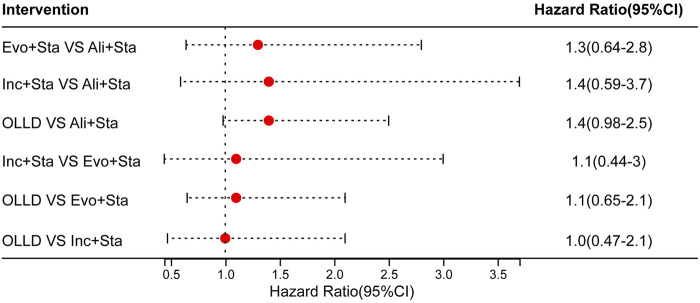
Ali: alirocumab; Evo: evolocumab; Inc: inclisiran; Sta:statins; OLLD: oral lipid-lowering drugs (statins, ezetimibe, or their combination).

In addition, subgroup analyses evaluating all-cause mortality showed that PCSK9 inhibitors added to statin treatment did not confer a significant mortality benefit. Comparisons among different PCSK9 inhibitor types also demonstrated no significant statistical differences in mortality rates (Figure 4).

### Safety results

**FIGURE 5:**
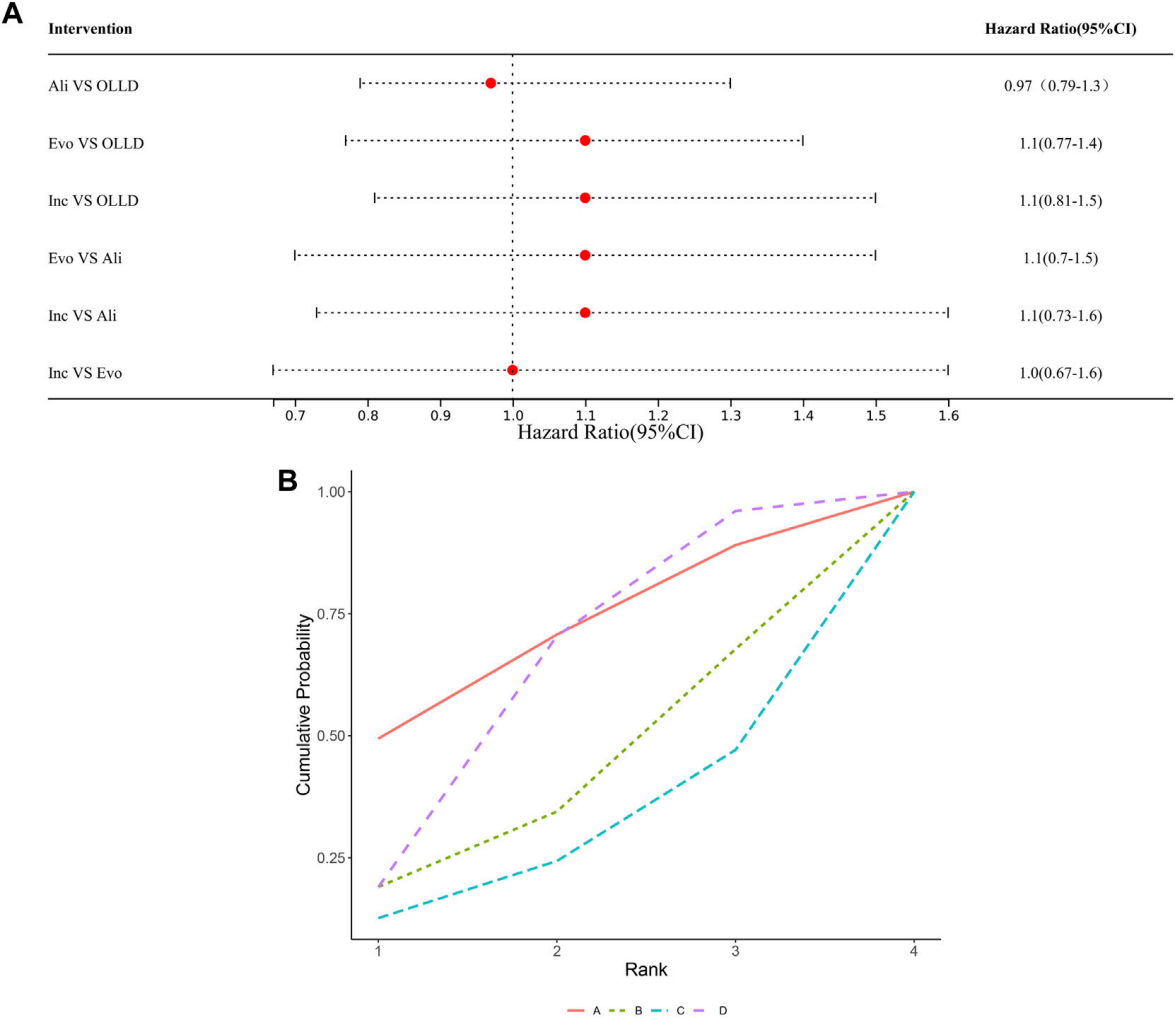
**(A)** Ali: alirocumab; Evo: evolocumab; Inc: inclisiran; OLLD: oral lipid-lowering drugs. **(B)** A: alirocumab; B: evolocumab; C: inclisiran; D oral lipid-lowering drugs.

**FIGURE 6:**
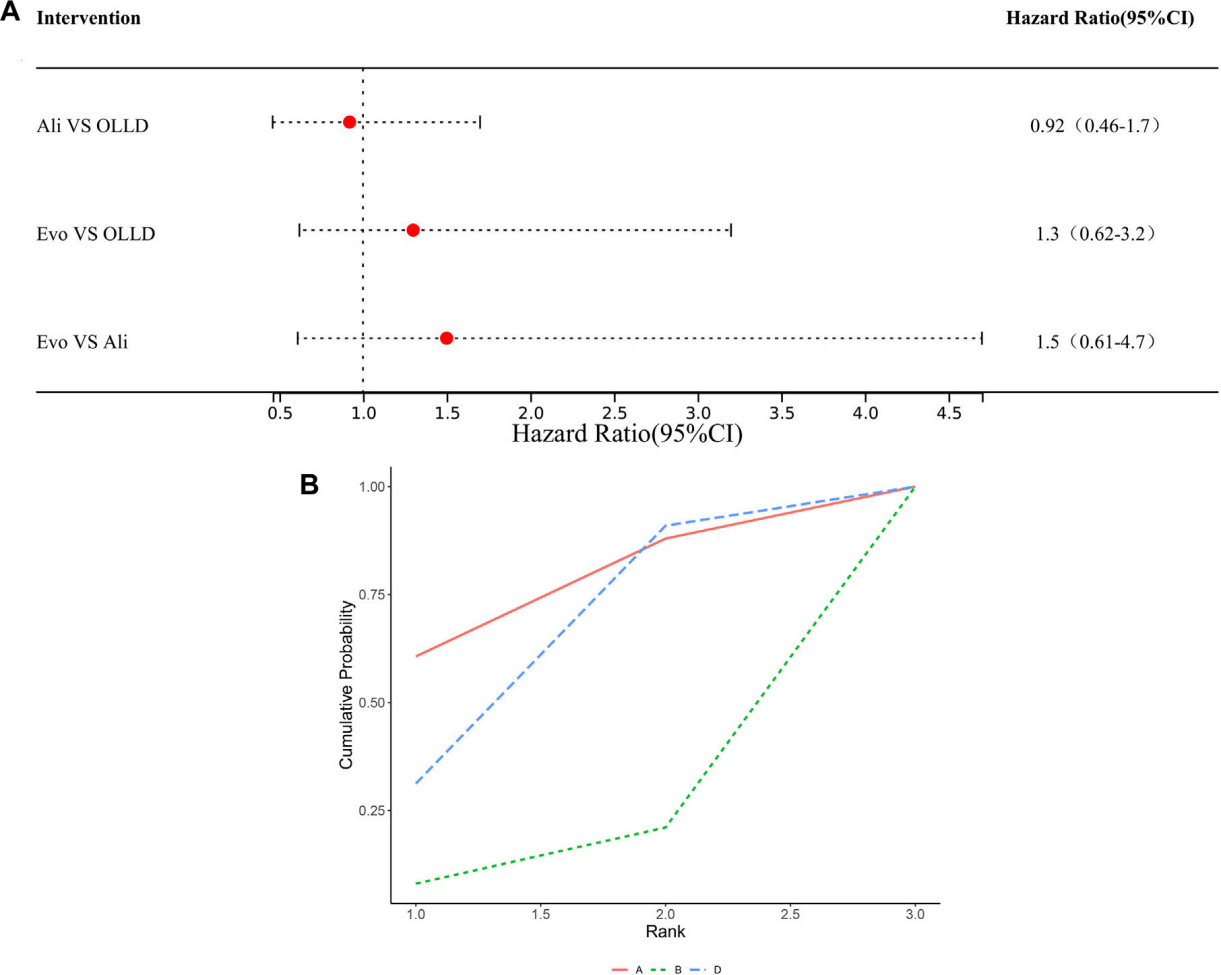
**(A)** Ali: alirocumab; Evo: evolocumab; OLLD: oral lipid-lowering drugs. **(B)** A: alirocumab; B: evolocumab; D: oral lipid-lowering drugs.

Regarding safety, the most notable concerns included two aspects: new-onset diabetes and neurocognitive impairment. A total of 19 studies in the literature reported adverse events related to new-onset diabetes and neurocognitive impairment. Among them, 10 studies reported adverse events related to new-onset diabetes, and 16 studies reported adverse events related to neurocognitive impairment. Compared to the control groups, there were no statistically significant differences in the risk of new-onset diabetes among various PCSK9 inhibitors (high certainty) (Figure 5A), ranked by SUCRA values as follows (Figure 5B): alirocumab (0.70), oral lipid-lowering drugs (0.62), evolocumab (0.4), and inclisiran (0.28). Similarly, no significant differences were found among treatment regimens in the incidence of neurocognitive impairment (data unavailable for inclisiran) (high certainty) (Figure 6A). Ranked by SUCRA values as follows (Figure 6B): alirocumab (0.74), oral lipid-lowering drugs (0.61), and evolocumab (0.15).

### Publication bias

**FIGURE 7:**
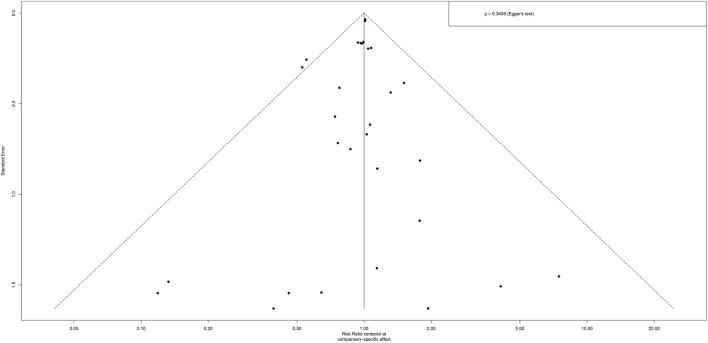
Funnel plot of network meta-analysis.

The funnel plot was approximately symmetrical (Figure 7), and the p value after Egger’s test was 0.3459, which indicated that there was no significant publication bias.

## Discussion

This study included a total of 29 RCTs aimed at evaluating various lipid-lowering regimens, including monotherapy with different PCSK9 inhibitors, combination therapy of different PCSK9 inhibitors with statins, and monotherapy with statins or ezetimibe, to assess their effectiveness in reducing MACE events and determining the optimal lipid-lowering regimen. According to our results, in terms of reducing MACE events, PCSK9 inhibitors combination with statins was significantly superior to other lipid-lowering regimens, among which Inclisiran combined with statins emerged with the highest probability of being the optimal choice based on SUCRA rankings. Additionally, as in the population included in the oral lipid-lowering drug group, more than 95% of the individuals used statins, our results indicate that the clinical benefits of statin monotherapy are not inferior to those of any PCSK9 inhibitor monotherapy.

Previous studies have consistently demonstrated the potent LDL-C lowering effects of statins and PCSK9 inhibitors (Sabatine et al., 2017; Schwartz et al., 2018; Raal et al., 2020; Cannon et al., 2004; LaRosa et al., 2005; Nissen et al., 2006). Statins inhibit cholesterol synthesis by limiting the action of HMG-CoA reductase, additionally, they upregulate LDL receptor (LDLR) expression and increase LDLR activity, enhancing the uptake and metabolism of LDL particles in the bloodstream. In contrast, PCSK9 inhibitors do not affect cholesterol synthesis directly. They primarily bind to free PCSK9, preventing it from attaching to LDL receptors (LDLRs), thereby reducing LDLR degradation and prolonging LDLR lifespan, which increases the clearance of LDL-C throughout its lifecycle (Norata et al., 2014; Roth and Davidson, 2018). Additionally, the efficacy of PCSK9 inhibitors in lowering LDL-C is limited by the concentration of free PCSK9. Once free PCSK9 levels reach zero, administering higher doses of PCSK9 inhibitors does not yield additional LDL-C reduction. Higher doses only prolong the duration of action without increasing the intensity of LDL-C lowering. It can be seen that statins and PCSK9 inhibitors have a synergistic effect in lowering LDL-C levels. Their combined use can further enhance lipid-lowering pathways, strengthen their respective mechanisms, and achieve a dual increase in the quantity and lifespan of LDLR. This leads to better LDL-C clearance capacity and significantly reduces LDL-C levels while providing additional clinical benefits by further lowering the risk of MACE. It’s consistent with our conclusions and aligns with the recommendations of various cardiovascular-related guidelines. Moreover, our subgroup analyses demonstrated that adding PCSK9 inhibitors to background statin therapy did not significantly reduce all-cause mortality compared to statin monotherapy. Additionally, no statistically significant differences were observed among various PCSK9 inhibitor agents regarding mortality outcomes. These findings suggest a consistent mortality profile across different PCSK9 inhibitors when used as adjunctive lipid-lowering therapy. This aligns with prior large-scale randomized controlled trials, which have similarly reported limited evidence for mortality reduction despite significant improvements in lipid parameters and major adverse cardiovascular event rates.

Current clinical paradigms regard PCSK9 inhibitors as novel potent lipid-lowering agents, which demonstrate enhanced LDL-C reduction efficacy compared to statins, thereby enabling superior clinical outcomes. However, as shown in Figure 2, the clinical benefits of statin monotherapy are not inferior to those of evolocumab and may even surpass those of alirocumab. This outcome surpassed our anticipated results. This may be due to the anti-inflammatory effects and improvement of endothelial function provided by statins beyond lipid-lowering (Liao and Laufs, 2005). Emerging evidence suggests that inflammation may play an even more pivotal role than dyslipidemia in the pathogenesis and progression of atherosclerosis (Ridker et al., 2023). The findings imply that the clinical benefits observed with certain interventions may not be solely attributable to their lipid-modifying effects. Rather, a more comprehensive mechanism—encompassing anti-inflammatory actions and improvements in endothelial function—may underlie the observed reduction in MACE risk. However, these effects are not yet well-documented for PCSK9 inhibitors, necessitating further research for confirmation. Furthermore, considering the factors of cost and administration route (Kazi et al., 2016; Khan et al., 2022), combined with treatment recommendations from both domestic and international guidelines, statins remain the most cost-effective and feasible monotherapy option for lipid lowering in most ASCVD patients requiring medication. Of course, the number of studies and participants in this subgroup was limited, and further research is warranted to validate these findings.

Additionally, among the three PCSK9 inhibitors currently widely used in clinical practice, the SCURA values indicate that the most beneficial treatment regimen may be the combination of inclisiran and statins. Of course, this result only comes from SUCRA rankings, without direct or statistical comparison supports. Based on the current evidence base, the optimal PCSK9 inhibitor cannot be definitively ascertained at present. Based on the SCURA ranking framework, we believe that this result may be due to inclisiran’s exceptionally long half-life, allowing it to maintain a stable low level of LDL-C in patients over a longer period, thereby reducing LDL-C fluctuations and minimizing the incidence and progression of ASCVD and MACE events. We keenly anticipates further evidence generation regarding inclisiran’s long-term clinical profile. However, due to cost considerations, its widespread use among ASCVD patients is currently limited, and we look forward to its promising performance once included in medical insurance coverage.

In terms of safety, we limited our discussion to newly diagnosed diabetes and neurocognitive impairment, as these outcomes were both clinically significant and sufficiently represented in our dataset. There is no statistically significant difference in the occurrence of new-onset diabetes or neurocognitive impairment. Several factors may explain this finding. First, most included trials investigated PCSK9 inhibitors as add-on therapy to established statin treatment, and the incremental risk for these adverse events may not substantially exceed that already associated with statins. Second, some trials reported a relatively low number of events and had limited follow-up durations, which could reduce the statistical power to detect rare adverse outcomes. Third, heterogeneity in diagnostic definitions and event reporting may have influenced the pooled estimates. Furthermore, these findings are consistent with the results of major outcome trials such as FOURIER and ODYSSEY OUTCOMES, which reported no significant increase in diabetes or neurocognitive adverse events with PCSK9 inhibitor therapy, even at very low LDL-C levels. Collectively, these observations suggest that while safety concerns warrant ongoing surveillance, current evidence does not indicate a substantial additional risk of diabetes or neurocognitive impairment when PCSK9 inhibitors are used on top of statins.

## Limitations

Our study has several limitations. Firstly, there is a lack of relevant studies directly comparing different types of PCSK9 inhibitors, and further large-scale studies are needed. Secondly, studies on monotherapy with PCSK9 inhibitors are limited, including the number of studies, number of participants, and follow-up duration. Thirdly, some studies may employ slightly different definitions of MACE, which may create bias.

## Conclusion

The emergence of PCSK9 inhibitors has provided us with more tools for lowering cholesterol, when combined with statins, it can provide the greatest clinical benefit for ASCVD patients. However, it is important to emphasize that statins remain the cornerstone of lipid-lowering therapy, with well-established clinical benefits. The long-term benefits of PCSK9 inhibitor monotherapy still require further clinical observation and validation. Therefore, when choosing monotherapy for lipid-lowering treatment, it is still recommended to first use statins in accordance with guideline recommendations.
